# P-640. Vancomycin *vs* Ceftaroline for Treatment of Methicillin-Resistant *Staphylococcus aureus* (MRSA) Pneumonia: A Multicenter Retrospective Study

**DOI:** 10.1093/ofid/ofae631.837

**Published:** 2025-01-29

**Authors:** Austin Auyeung, Sanil Thomas

**Affiliations:** University of Central Florida College of Medicine/HCA Florida North Florida Hospital, Gainesville, Florida; HCA Florida North Florida Hospital, Gainesville, Florida

## Abstract

**Background:**

Vancomycin (V) is considered first-line treatment for MRSA infections, assuming bacterial susceptibility and patient tolerability. Ceftaroline (C) has been used off-label to treat MRSA blood stream infections, osteomyelitis, ventilator-associated pneumonia, hospital-acquired pneumonia and endocarditis. However, the efficacy of C vs V for treatment of MRSA pneumonia has not been studied.

**Figure 1.**
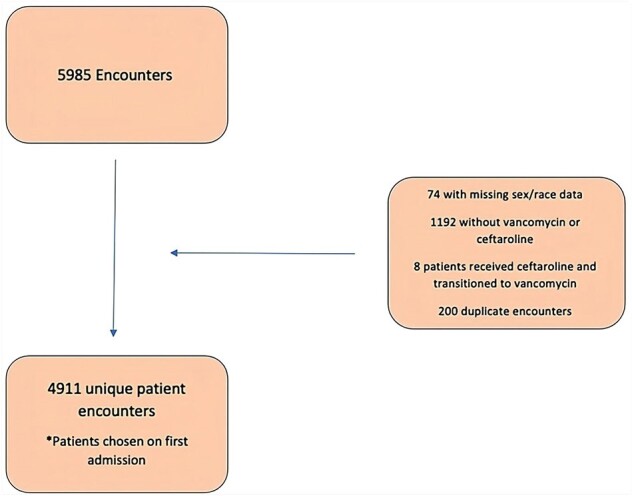
Summary of Exclusions for Patient Population

**Methods:**

Data was extracted from a centralized database for 184 HCA Healthcare hospitals from January 1^st^, 2016 – June 30^th^, 2023. Patient encounters with a diagnostic code of MRSA pneumonia and received V and/or C were included for further analysis. In-hospital mortality and length of stay (LOS) were the main outcomes studied. Demographics including age, sex and race were extracted. Average vitals values noted included temperature and O_2_ saturation. Lab values included average WBC count, creatinine and GFR. Time to first dose of V or C from time of admission and time to transition from V to C were included. Patients who received C-only were combined with the patients who first received V and then transitioned to C (VC). IRB approval was obtained prior to data extraction.

**Table 1.**
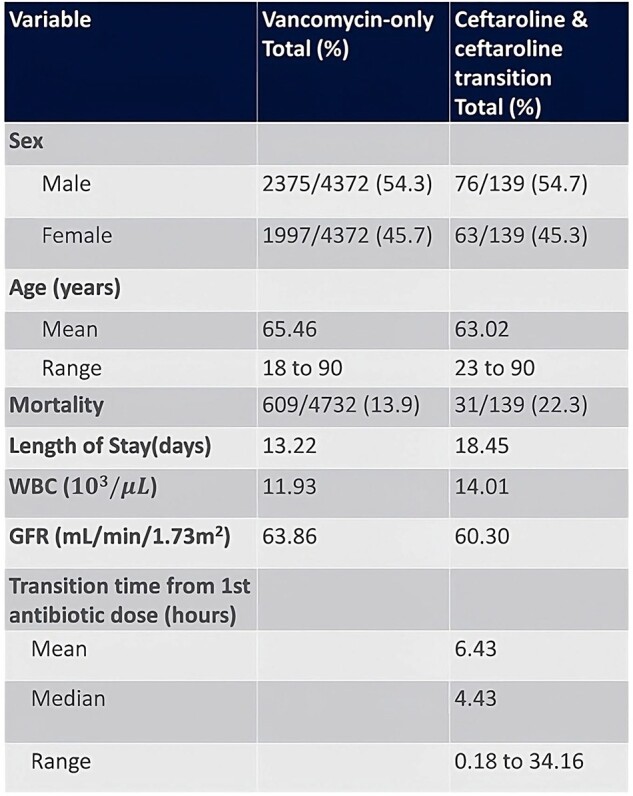
Demographics, Clinical Data and Outcomes

**Results:**

Initial data extraction yielded 5977 encounters with final analysis for 4511 unique patients. Encounters were excluded for missing demographic data, duplicates or when V or C were not received. The V-only group had 4372 encounters and the C+VC combined group had 139 encounters.

The mortality rate in the V-only group was 609/4372 (13.9%), while that in the C+VC group was 31/139 (22.3%). LOS was 13.22 days and 18.45 days in the V only and C+VC groups, respectively.

The average time to transition from V to C in the VC group was 6.4 hours with maximum of 34 hours.

Average temperature and O_2_ saturation were not significantly different between the two groups. GFR (mL/min/1.73m^2^) was 63.86 and 60.30 and WBC (10^3^/µL) count was 11.93 and 14.01 in the V and V+VC groups, respectively.

**Conclusion:**

Preliminary data suggest that V may be superior to C for MRSA pneumonia with respect to mortality and hospital LOS. Average WBC may be greater in the C+VC group, suggesting more severe illness. Reasons for transition of V to C were unknown, however given the short transition time, the impact of V on treatment outcome is likely negligible.

**Disclosures:**

**All Authors**: No reported disclosures

